# Supplementary material for: USP48 restrains resection by site-specific cleavage of the BRCA1 ubiquitin mark from H2A
Source: Nat Commun. 2018 Jan 15;9:229. doi: 10.1038/s41467-017-02653-3 (PMC5768779; doi:10.1038/s41467-017-02653-3)
Supplement: Supplementary file 1 — Supplementary Information [file 41467_2017_2653_MOESM1_ESM.pdf]

Supplementary Table 1

Best fit values for Michaelis-Menten kinetics from Figure S1A.

**Michaelis-Menten kinetics**			
	**Kcat (s^{-1})**	**Km (uM)**	**Kcat/Km (s^{-1} uM^{-1})**
**USP1/UAF1**	1.18 ± 0.06	0.34 ± 0.06	3.45
**USP3**	0.00360 ± 0.00006	6.29 ± 0.21	0.0006
**USP7**	0.51 ± 0.04	17.50 ± 2.52	0.029
**USP11**	0.020 ± 0.001	0.31 ± 0.03	0.076
**USP12/UAF1**	0.064 ± 0.002	4.31 ± 0.45	0.079
**USP15**	0.23 ± 0.02	1.61 ± 0.21	0.143
**USP16**	0.26 ± 0.006	8.20 ± 0.49	0.032
**$USP48^{Iso1}$**	0.35 ± 0.014	61.31 ± 4.13	0.004
**$USP48^{Iso2}$**	0.02 ± 0.0007	5.14 ± 0.53	0.006
**BAP1/ASXL1**	1.78 ± 0.031	3.57 ± 0.19	0.499

Supplementary Table 2

Oligonucleotide primers used for cloning and site-directed mutagenesis

**Cloning Primers**	
**Cloning USP48^{Iso1}**	
USP48 EcoRI Fwd	GTT GGG AAT TCT GCT CTT TTG TGT CCC CAC GGG
USP48 EcoRI Rev	TGA TGA CAC AGG GCT GC
USP48 iso1 gBlock	CCTTGAATTCTGCTCTTTTGTGTCCCCACGGGGGCCTCATGTTTACATTTGCTTCCATGACCAA<br>AGAAGATTCTAAACTTATAGCTCTCATATGGCCCAGTGAGTGGCAAATGATACAAAAGCTCTT<br>TGTTGTGGATCATGTAATTAAAATCACGAGAATTGAAGTGGGAGATGTAAACCCTTCAGAAA<br>CACAGTATATTTCTGAGCCCAAACTCTGTCCAGAATGCAGAGAAGGCTTATTGTGTCAGCAGC<br>AGAGGGACCTGCGTGAATACACTCAAGCCACCATCTATGTCCATAAAGTTGTGGATAATAAA<br>AAGGTGATGAAGGATTCGGCTCCGGAACTGAATGTGAGTAGTTCTGAAACAGAGGAGGACA<br>AGGAAGAAGCTAAACCAGATGGAGAAAAAGATCCAGATTTTAATCAAAGCAATGGTGGAAC<br>AAAGCGGCAAAAGATATCCCATCAAAATTATATAGCCTATCAAAAGCAAGTTATTCGCCGAA<br>GTATGCGACATAGAAAAGTTCGTGGTGAGAAAGCACTTCTCGTTTCTGCTAATCAGACGTTAA<br>AAGAATTGAAAATTCAGATCATGCATGCATTTTCAGTTGCTCCTTTTGACCAGAATTTGTCAAT<br>TGATGGAAAGATTTTAAGTGATGACTGTGCCACCCTAGGCACCCTTGGCGTCATTCCTGAATC<br>TGTCATTTTATTGAAGGCTGATGAACCAATTGCAGATTATGCTGCAATGGATGATGTCATGCA<br>AGTTTGTATGCCAGAAGAAGGGTTTAAAGGTACTGGTCTTCTTGGACATTAACCGGGCTTCTC<br>CTCGAGAAGG
**Mutagenesis Primers**	
**USP48**	
USP48_C98S_F	CCTTGGAGCCACTTcTTATGTCAACAC
USP48_C98S_R	GTGTTGACATAAgAAGTGGCTCCAAGG
USP48_siR_Ex5_F	GTCTAAACAAAAGAATCCtGAcGTcaGgAATATTGTTCAACAGCAG
USP48_siR_Ex5_R	CTGCTGTTGAACAATATTcCtgACgTCaGGATTCTTTTGTTTAGAC
USP48_siR_Ex11_F	GGCTGAGATGCGTAAaCAgtcaGTcGATAAAGGAAAAG
USP48_siR_Ex11_R	GCTTTTCCTTTATCgACtgacTGtTTACGCATCTCAGCC

Supplementary Table 3

Details of siRNA sequences

siRNA sequences	
NTC<br>(Renilla Luciferase)	Sense: CUUACGCUGAGUACUUCGA[dT][dT]<br>Antisense: [Phos]UCGAAGUACUCAGCGUAA G[dT][dT]
USP48 Exon 5	Sense: GCGUAAGCAAAGUGUGGAUAA[dT][dT]<br>Antisense: [Phos]UUAUCCACACUUUGCUUACGC[dT][dT]
USP48 Exon 11	Sense: GAAUCCAGAUGUGCGCAAUAU[dT][dT]<br>Antisense: [Phos]AUAUUGCGCACAUCUGGAUUC[dT][dT]
murine USP48-1	Sense: AUUCCUUUGUGGGCUUGACUA[dT][dT]<br>Antisense: [Phos]UAGUCAAGCCCACAAAGGAAU[dT][dT]
murine USP48-2	Sense: AUUCUGGCCACUACAUCGCAC[dT][dT]<br>Antisense: [Phos]GUGCGAUGUAGUGGCCAGAAU[dT][dT]
53BP1 smartpool	Dharmacon - Product Code L-003548-00-0005
BARD1	Sense: UGGUUUAGCCCUCGAAGUAAG[dT][dT]<br>Antisense: [Phos]CUUACUUCGAGGGCUAAACCA[dT][dT]
BRCA1 3’UTR 1	Sense: GCUCCUCUCACUCUUCAGU[dTdT]<br>Antisense: [Phos]ACUGAAGAGUGAGAGGAGC[dT][dT]
BRCA1 3’UTR 2	Sense: AAGCUCCUCUCACUCUUCAGU[dT][dT]<br>Antisense: [Phos]ACUGAAGAGUGAGAGGAGCUU[dT][dT]
CtIP	Sense: GGACCUUUGGACAAAACUAAA [dT][dT]<br>Antisense: [Phos]UUUAGUUUUGUCCAAAGGUCC[dT][dT]
RAD51 Exon 9	Sense: CCCUUUACAGAACAGACUA[dT][dT]<br>Antisense: [Phos]UAGUCUGUUCUGUAAAGGG[dT][dT]
RAD51 Exon 11	Sense: UGAAGCUAUGUUCGCCAUU[dT][dT]<br>Antisense: [Phos]AAUGGCGAACAUAGCUUCA[dT][dT]
RAD52 Exon 6	Sense: CGGGUAAUUAAUCUGGCCAAU[dT][dT]<br>Antisense: [Phos]AUUGGCCAGAUUAAUUACCCG[dT][dT]
RAD52 Exon 14	Sense: CCACCAGAAACCACAAGCAAA[dT][dT]<br>Antisense: [Phos]UUUGCUUGUGGUUUCUGGUGG[dT][dT]
SMARCAD1 #1	Sense: GACGAUUGAAGAAUCCAUGCU [dTdT]<br>Antisense: [Phos]AGCAUGGAUUCUUCAAUCGUC[dTdT]
SMARCAD1 #2	Sense: AUGUAGUUAUAAGGCUUAUGA[dTdT]<br>Antisense: [Phos]UCAUAAGCCUUAUAACUACAU[dTdT]

Supplementary Table 4

Details of antibodies and concentrations

Antibody	Animal	Supplier	Cat. number	Technique	Conc
53BP1	Goat	R&D Systems	Af1877	IF	1:5000
53BP1	Rabbit	Abcam	Ab36823	WB	1:5000
β-actin	Rabbit	Abcam	Ab8227	WB	1:3000
BRCA1 (D9)	Mouse	Santa Cruz	Sc6954	IF	1:500
BRCA1 (MS110)	Mouse	Calbiochem	OP94	WB	1:500
BrdU	mouse	Becton Dickinson	347580	Resection fibres	1:500
CtIP	Rabbit	Abcam	ab155988	WB	1:500
Flag	Mouse	Sigma	F1804	WB	1:1000
HA	Mouse	Sigma	H336	WB	1:1000
RAD51	Rabbit	Santa Cruz	SC8349	IF	1:1000
RAD52	Sheep	*Gift from Fena Ochs/Claudia Lukas*		WB	1:1000
RPA	Mouse	Abcam	Ab2175	IF	1:2000
SMARCAD1	Rabbit	Bethyl	a301-593a-m	WB	1:1000
USP48	Rabbit	Abcam	Ab72226	WB/IF	1:1000
Donkey α Mouse AlexaFluor 488	Donkey	Life technologies	A21202	IF	1:5000
Donkey α Rabbit AlexaFluor 555	Donkey	Life technologies	A31572	IF	1:5000
Donkey α Goat AlexaFluor 488	Donkey	Life technologies	A11055	IF	1:5000
Rabbit α Mouse HRP	Rabbit	Dako	P0161	WB	1:10000
Swine α Rabbit HRP	Swine	Dako	P0217	WB	1:10000
Rabbit α Goat HRP	Rabbit	Dako	P0449	WB	1:10000

Supplementary Figure 1

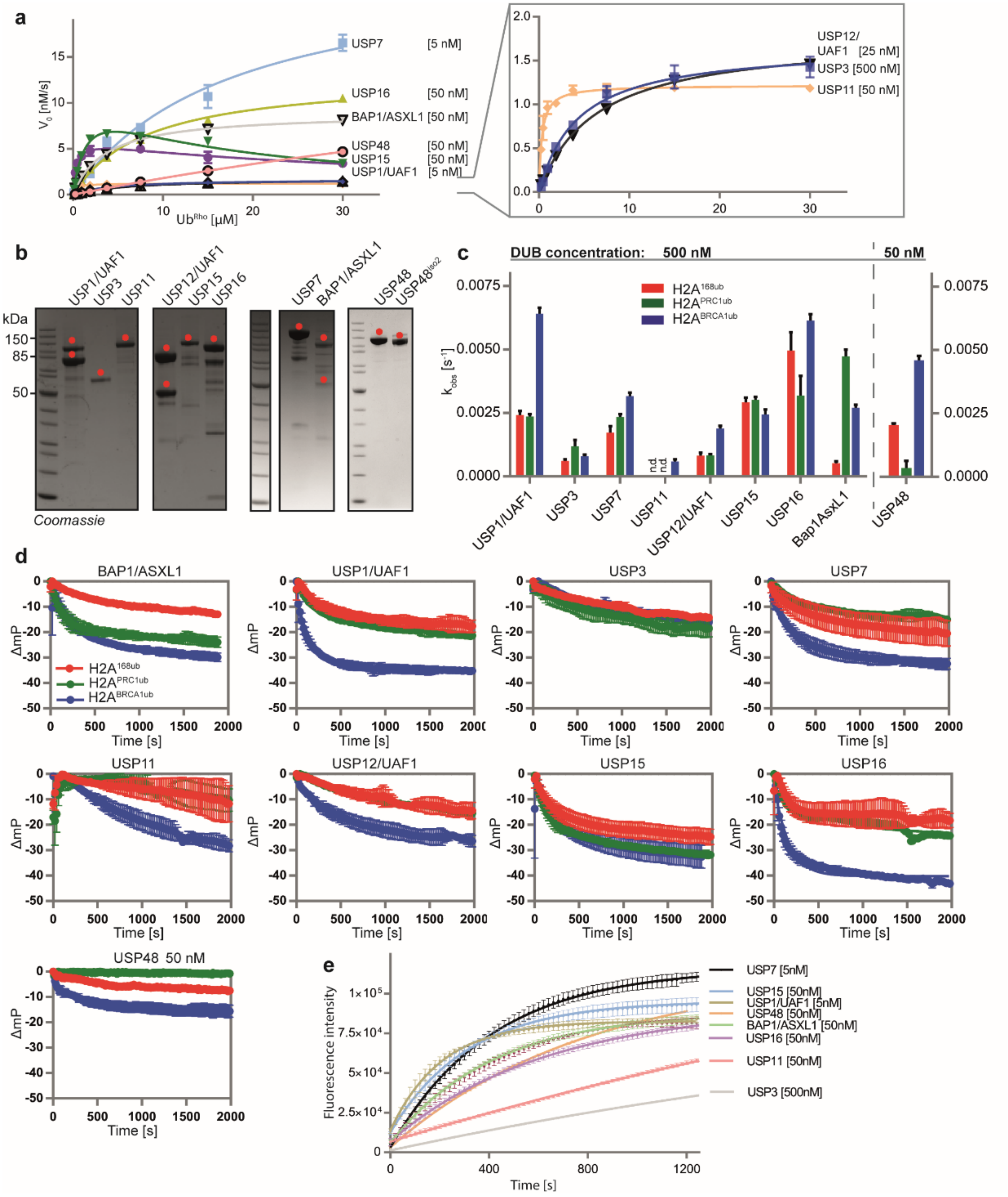

Supplementary Figure 1
DUB activity screening data

a Michaelis-Menten kinetics of the purified DUBs on minimal substrate Ub^{Rho}. Different enzyme concentrations were used for different DUBs as indicated in the figure. The speed of the linear phase of the reaction is plotted at different substrate concentrations. The data were fit to the Michaelis-Menten equation using the program GraphPad Prism. See also Supplementary Table 1. Replicates of two experiments ± s.e.m

b Purified DUBs used on in this study. * Indicates bands corresponding to the respective DUB

c DUBs tested show no exclusive site specificity but rather preferential cleavage of $H2A^{168ub}$, $H2A^{PRC1ub}$ and $H2A^{BRCA1ub}$ in NCPs. Site specific reaction speed quantified for all DUBs tested. k_{obs} values were obtained by fitting an exponential function to the traces in D). Replicates of two experiments ± s.e.m.

d Raw data of the FP assay to identify site specific DUBs. 2 µM of $H2A^{168ub}$, $H2A^{PRC1ub}$ or $H2A^{BRCA1ub}$ were cleaved by 500 nM of the indicated DUB. Different amplitudes are because the distance from the center of mass to the ubiquitination sites is different for differently modified NCP. Replicates of two experiments ± s.e.m

e Activity of the DUBs included in this study on minimal substrate. Cleavage of 2 µM minimal substrate Ub^{Rho} using the same conditions as in the FP screen on nucleosomal substrates. Concentrations of the respective DUB used are indicated.

Supplementary Figure 2

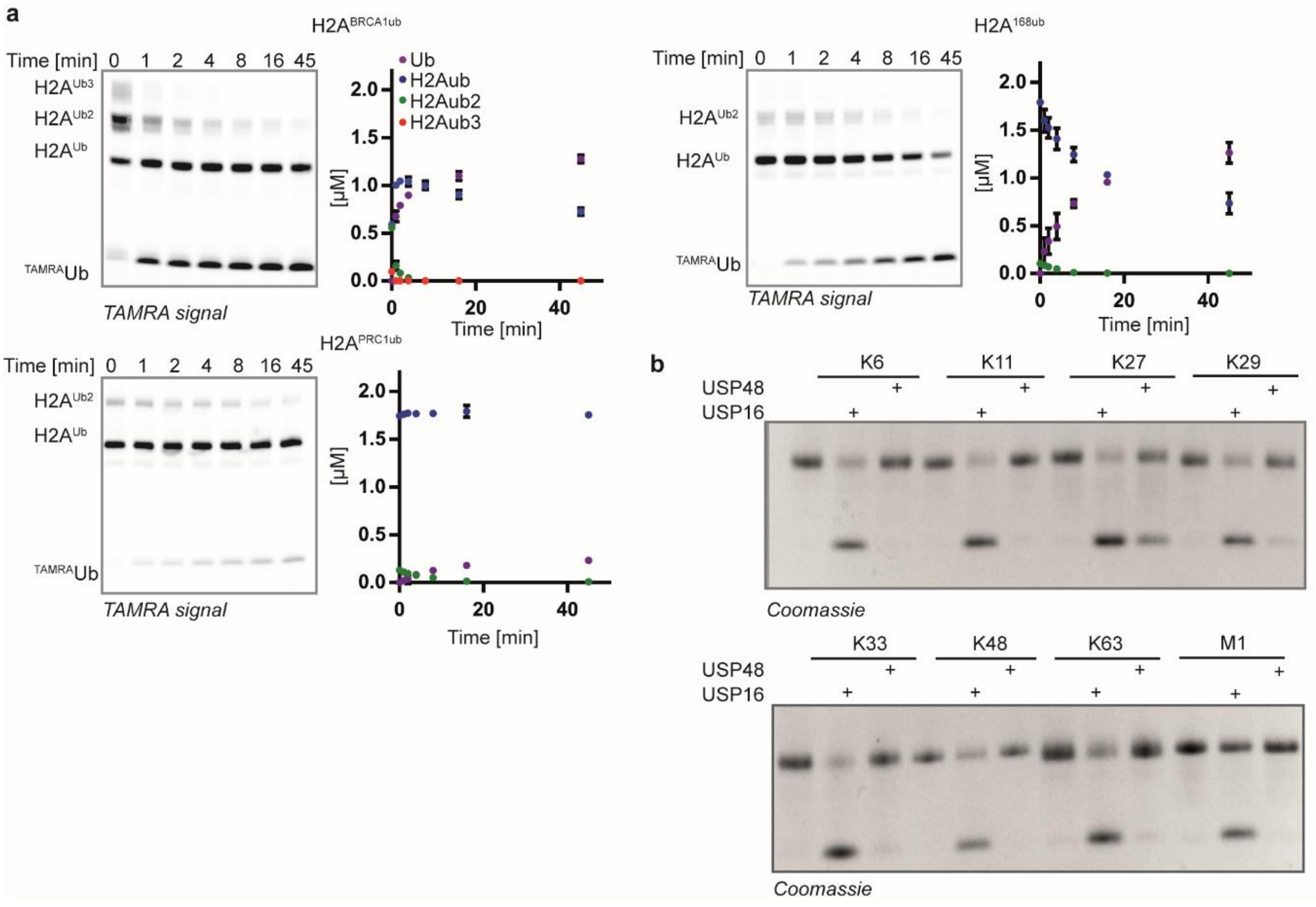

Supplementary Figure 2

Characterization of USP48 site specificity

a Time course of cleavage of 2 µM H2A^{168ub} , H2A^{PRC1ub} and H2A^{BRCA1ub} in NCPs by USP48 (50 nM) on SDS-gel quantified using ^{TAMRA}Ub. Fluorescent gel scans and quantification of the individual reaction species are shown. Replicates of two experiments ± s.e.m

b Cleavage of all the di-Ubiquitin linkages by USP48 (100 nM). USP16 (500 nM) serves as an unspecific positive control.

Supplementary Figure 3

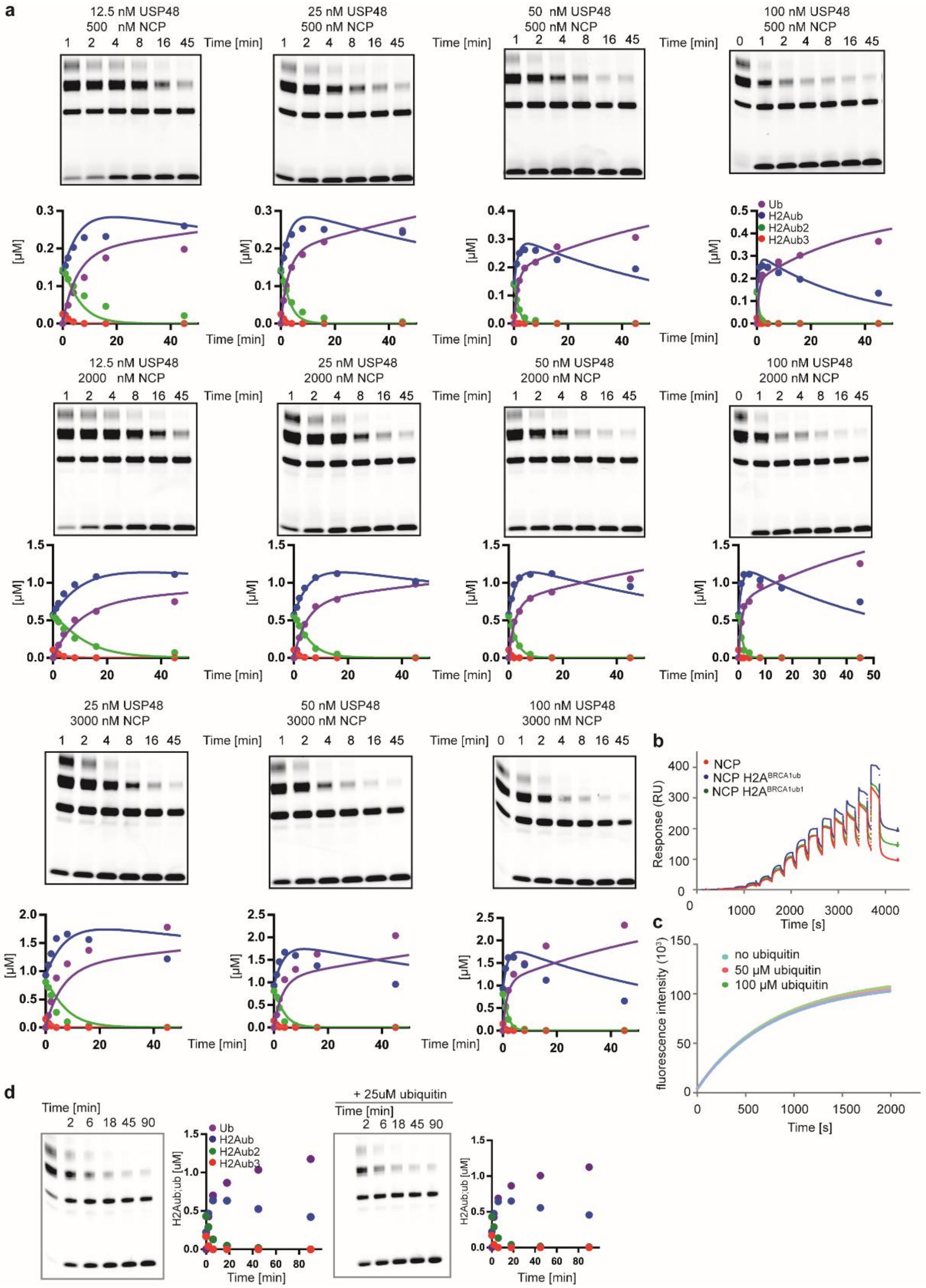

Supplementary Figure 3

Kinetic analysis of USP48 cleavage of H2A^{BRCA1ub}

a Gel based cleavage assay of H2A^{BRCA1ub} kinetic analysis in Figure 2. ^{TAMRA}Ub was used as readout and concentration of USP48 ^{iso1} and H2A^{BRCA1ub} were varied across the range indicated. Fluorescence readout of the gels and quantifications are shown including the fit obtained from fitting with *KinTek explorer*.

b USP48 binding to NCPs of different ubiquitination status. Raw traces from the SPR experiments fitted in Figure 2d.

c Ubiquitin does not activate USP48 ^{iso1}. Cleavage of 2 µM Ub^{Rho} by 50 nM USP48 ^{iso1}in the presence of indicated ubiquitin concentrations.

d USP48^{iso2} cleavage of 2 µM H2A^{BRCA1ub} in the absence and presence of 25 µM ubiquitin.

Supplementary Figure 4

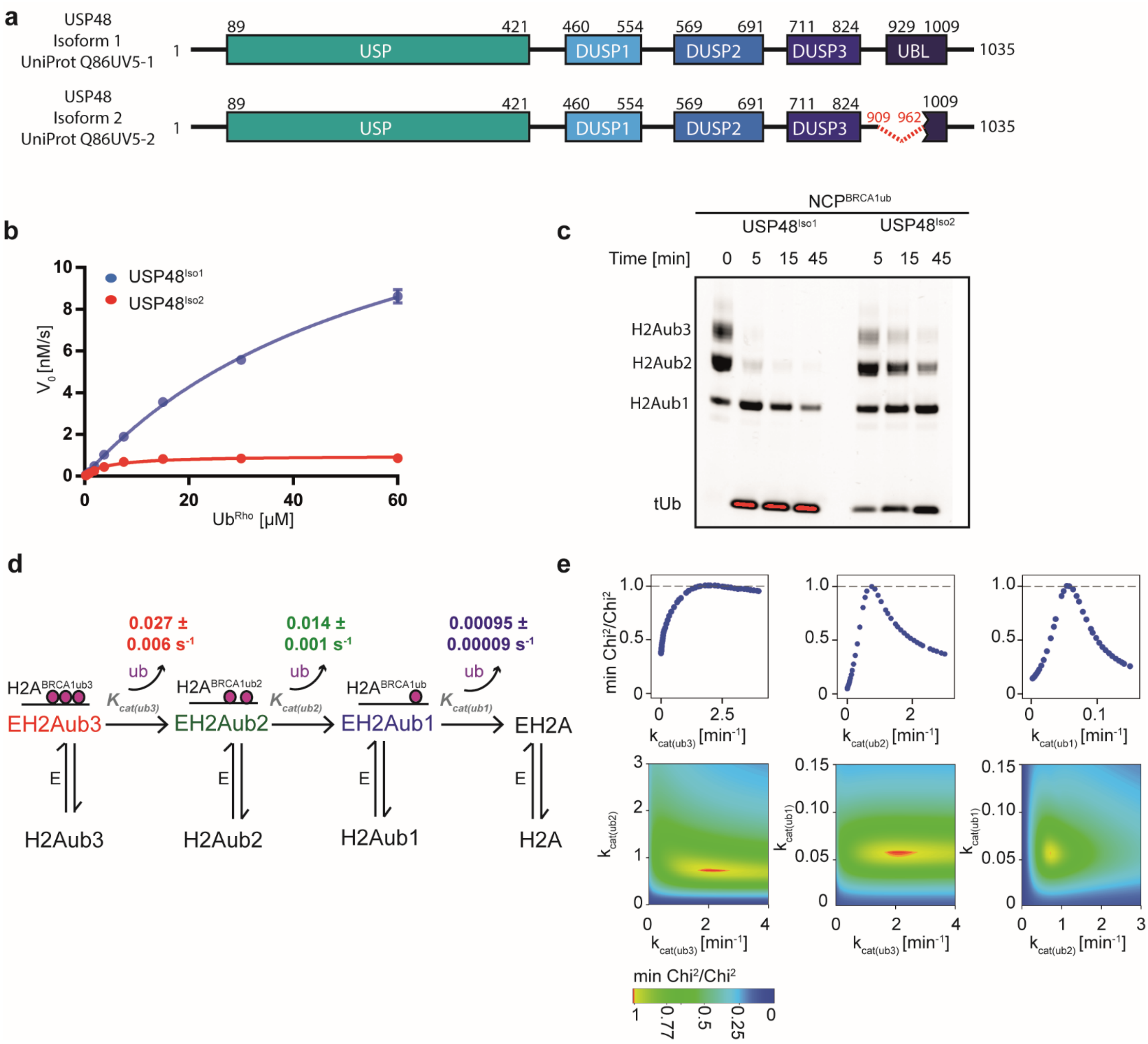

Supplementary Figure 4

Comparison between USP48^{Iso1} and USP48^{Iso2} kinetics

a Schematic representation of USP48 isoform 1 and 2.

b Full length, USP48^{Iso1} has higher activity on minimal substrate Ub^{Rho} compared to USP48^{Iso2}. Cleavage of different UbRho concentrations by 50 nM USP48 (isoform1 or isoform 2). Data were fitted to a standard Michalis-Menten model using *GraphPad Prism*.

c USP48 ^{iso1} is more active than USP48 ^{iso2} on nucleosomal substrates. Cleavage of 2 µM NCP^{BRCA1ub} ubiquitinated with ^{TAMRA}Ub by 1 µM of USP48 (isoform1 or isoform 2). Overexposed pixel are indicated in red.

d USP48^{iso2} cleaves H2A^{BRCA1ub} in nucleosomes 15-30 times faster when the auxiliary ubiquitin is present. Kinetic model describing USP48's cleavage pattern on H2A^{BRCA1ub} with the fitted values for $k_{cat(ub3)}$, $k_{cat(ub2)}$ and $k_{cat(ub1)}$ and the standard error of the fit.

e Parameters for $k_{cat(ub3)}$, $k_{cat(ub2)}$ and $k_{cat(ub1)}$ in **d** are well constrained by the data. Evaluation of the goodness of fit. The upper three panels show how well defined the lower and upper boundaries are for the individual parameters. The lower panel shows how Chi^2 varies when two of the fitted variables are varied against each other. Red indicates a Chi^2 minimum.

Supplementary Figure 5

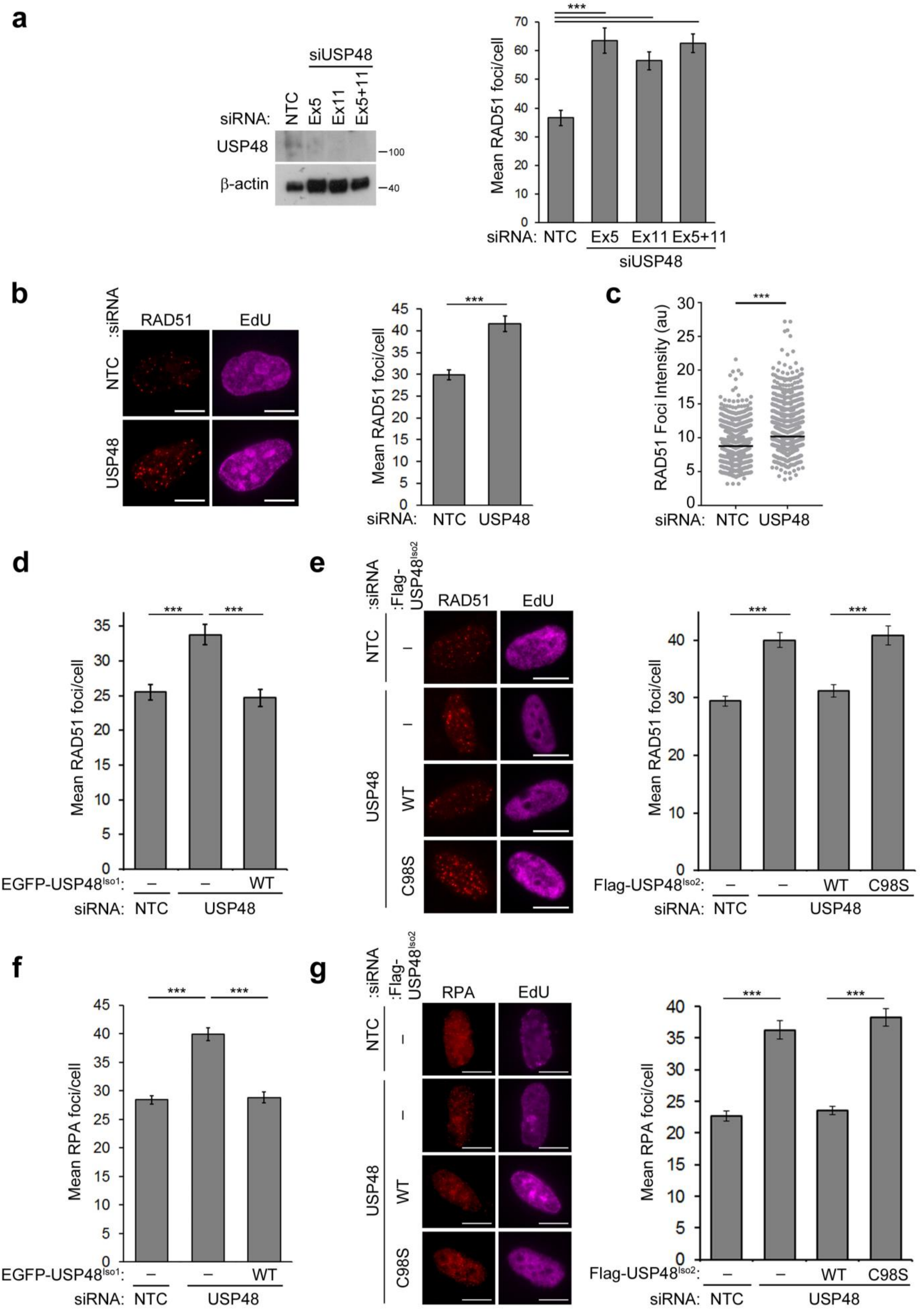

Supplementary Figure 5

siResistant USP48$^{Iso1/Iso2}$ prevents the increase in RAD51 and RPA foci observed following USP48 depletion

a. USP48 knockdown increases RAD51 foci formation. Left hand image shows western blot for USP48 expression levels in HeLa cells treated with two independent siRNA sequences against exon 5 (Ex5) and exon 11 (Ex11) either treated independently or together and cells treated with non-targeting control siRNA (NTC). The right hand graph shows quantification of RAD51 foci in S-phase (EdU positive) HeLa cells similarly treated with siRNA sequences and fixed at 2 hours post 5 Gy IR. Graph shows mean RAD51 foci/cell, n >30 cells, errors = s.e.m. *** p<0.005 Student's T-test.

b. Left hand panel shows images of RAD51 foci in S-phase (EdU positive) U20S cells depleted for USP48 (Ex5+Ex11) or NTC siRNA and fixed at 2 hours post 5 Gy IR. Scale bar 10 μm. Graph shows quantification of mean RAD51 foci/cell, n=100 cells, errors = s.e.m. *** p<0.005 Student's T-test.

c. Quantification of RAD51 foci intensity (arbitrary units (au)) in S-phase HeLa cells depleted for USP48 and fixed at 2 hours post 5 Gy IR. Graph shows mean RAD51 foci intensity (Foci counted from 115 cells: n=985 foci NTC, n=1170 foci USP48), errors = s.e.m *** p<0.005, ns=non-significant, Student's T-test.

d-e. Expression of EGFP-USP48^{Iso1}-WT, n=90 cells (**d**) or Flag-USP48^{Iso2}-WT but not Flag-USP48^{Iso2}-C98S, n=225 cells (**e**) restores lower numbers of RAD51 foci to USP48-depleted cells. RAD51 foci were measured in S-phase (EdU positive) cells fixed 2 hours post-5 Gy IR. Graph shows mean RAD51 foci/cell, errors = s.e.m *** p<0.005 Student's T-test. Scale bars 10 μm.

f-g. Expression of EGFP-USP48^{Iso1}-WT, n=130 cells (**f**) or Flag-USP48^{Iso2}-WT but not Flag-USP48^{Iso2}-C98S, n=174 cells (**g**) restores lower numbers of RPA foci to USP48-depleted cells. Cells were fixed at 2 hours post-5 Gy IR and RPA foci measured in S-phase (EdU positive) cells. Graph shows mean RPA foci/cell, errors = s.e.m *** p<0.005 Student's T-test. Scale bars 10 μm.

Supplementary Figure 6

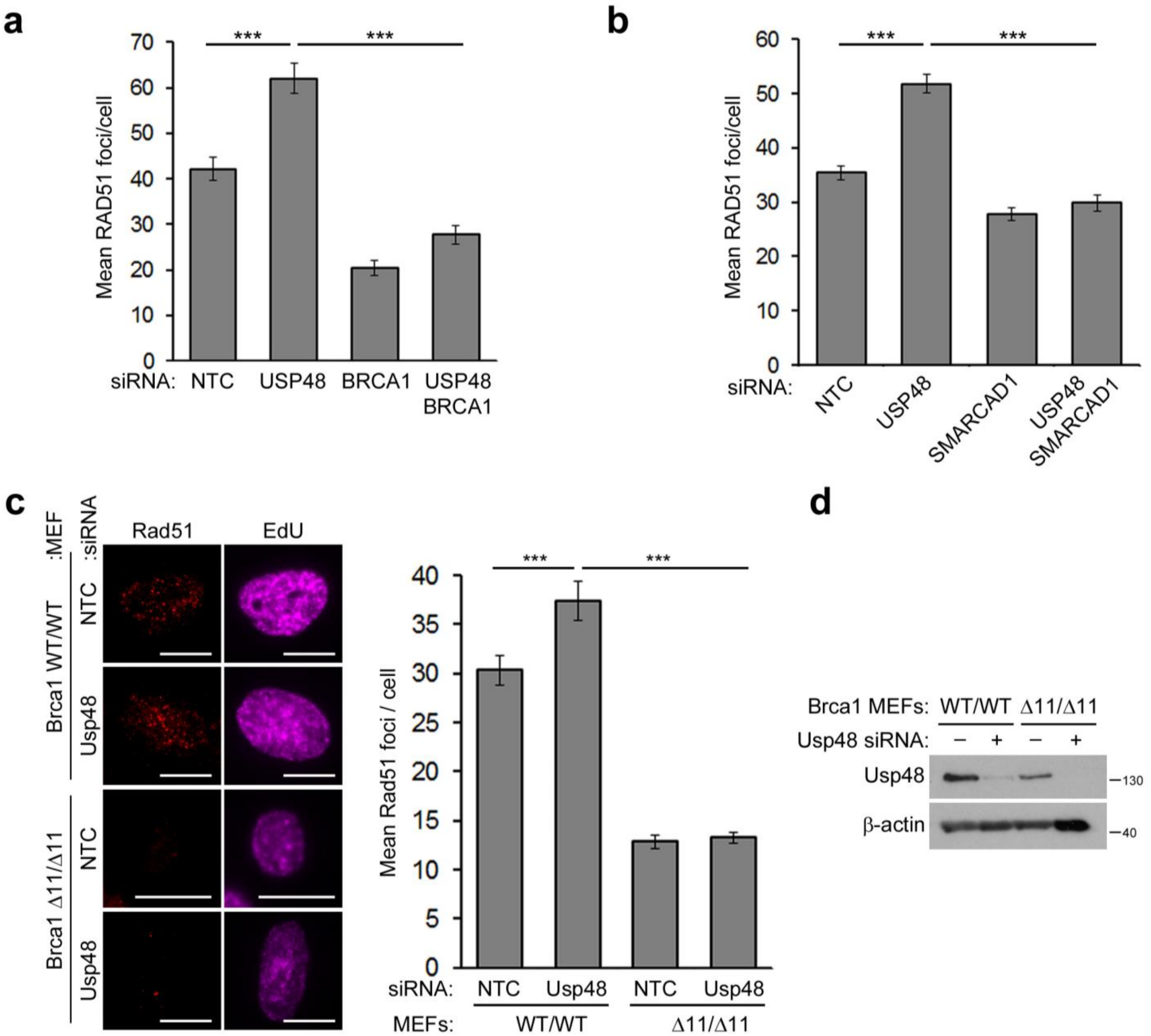

Supplementary Figure 6

USP48 acts in the same pathway as BRCA1 and SMARCAD1 for RAD51 foci formation

a & b. Quantification of RAD51 in S-phase (EdU positive) HeLa cells depleted for (**a**) USP48 and BRCA1 (n=60 cells) or (**b**) USP48 and SMARCAD1 (n=145 cells). Cells were fixed at 2 hours post-5 Gy irradiation. Mean RAD51 foci/cell, errors = s.e.m *** p<0.005 Student's T-test.

c. Depletion of murine Usp48 increases Rad51 foci numbers in control but not Brca1-mutant cells. Rad51 foci were measured in S-phase (EdU positive) *WT* and $Brca1^{\Delta11/\Delta11}$ MEFs depleted for Usp48 or treated with non-targeting control siRNA (NTC). Cells were fixed at 2 hours post-5 Gy irradiation and stained for Rad51. Left hand panel shows representative images, Scale bars 10 µm. Right hand graph shows mean Rad51 foci/cell, n=100 cells, errors = s.e.m *** p<0.005 Student's T-test.

d. Western blots to demonstrate protein expression of murine Usp48 in *WT* and $Brca1^{\Delta11/\Delta11}$ MEFs treated with Usp48 siRNA.

Supplementary Figure 7

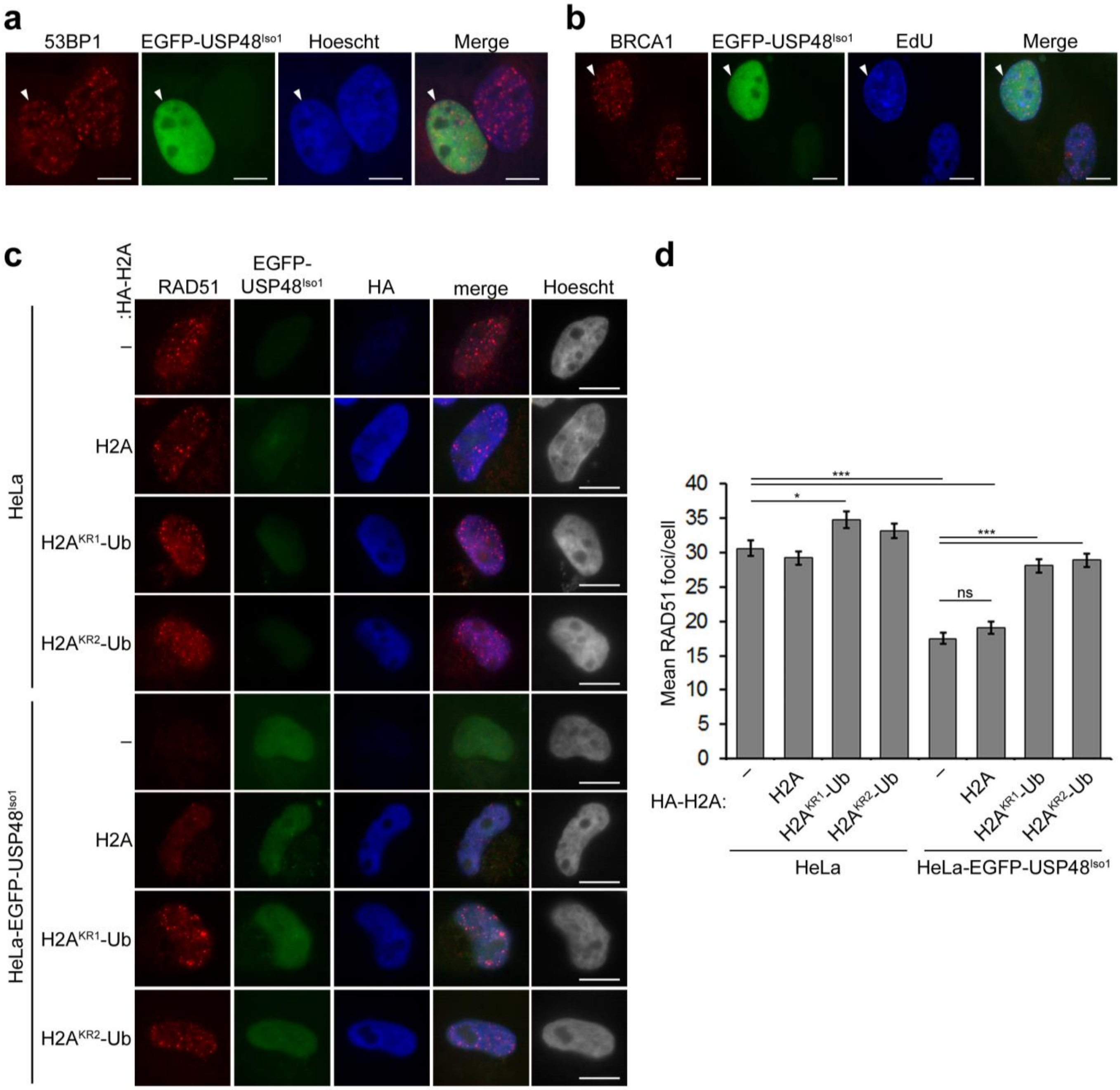

Supplementary Figure 7

The effects of USP48^{Iso1} overexpression can be rescued by a C-terminal H2A-Ub fusion mimic

a & b. No impact of EGFP-USP48^{Iso1} expression on BRCA1 or 53BP1 foci formation. HeLa FlpIn EGFP-USP48^{Iso1} cells were treated with doxycycline to induce expression for 48 hours before addition of EdU and irradiation with 5 Gy. Cells were fixed in 4% PFA, 1 hr post irradiation before staining with antibodies to 53BP1 (a) or BRCA1 (b). HeLa cells expressing Flag-USP48^{Iso2} were similarly evaluated (not shown). USP48^{Iso2} is largely cytoplasmic but not exclusively so, with both nuclear and cytoplasmic localisation seen in a small percentage of cells. Similar to isoform 1, ectopic expression of isoform 2 has no impact on 53BP1 or BRCA1 foci formation after IR.

c & d. Ectopic expression of EGFP-USP48^{Iso1} represses RAD51 foci formation, which can be rescued by expression of an H2A~Ub fusion protein. Wild-type HA-tagged-H2A, or one of two forms of H2A genetically fused to K-less Ubiquitin, were transfected into HeLa FlpIn EGFP-USP48^{Iso1} cells treated with doxycycline. In the fusion construct H2A^{KR1}-Ub, H2A lysines 125, 127 and 129 were mutated to arginine; in H2A ^{KR2}-Ub, H2A lysines 13/15, 118/119 and 125,127, 129 were replaced with arginine. Images (left) show RAD51 foci in control or EGFP-USP48^{Iso1} cells, with or without ectopic HA-H2A expression (HA), fixed at 2 hours post 5 Gy IR. Graph (right) shows mean RAD51 foci/cell, n=100 cells, errors = s.e.m * $p<0.05$, *** $p<0.005$, ns=non-significant, Student's T-test.

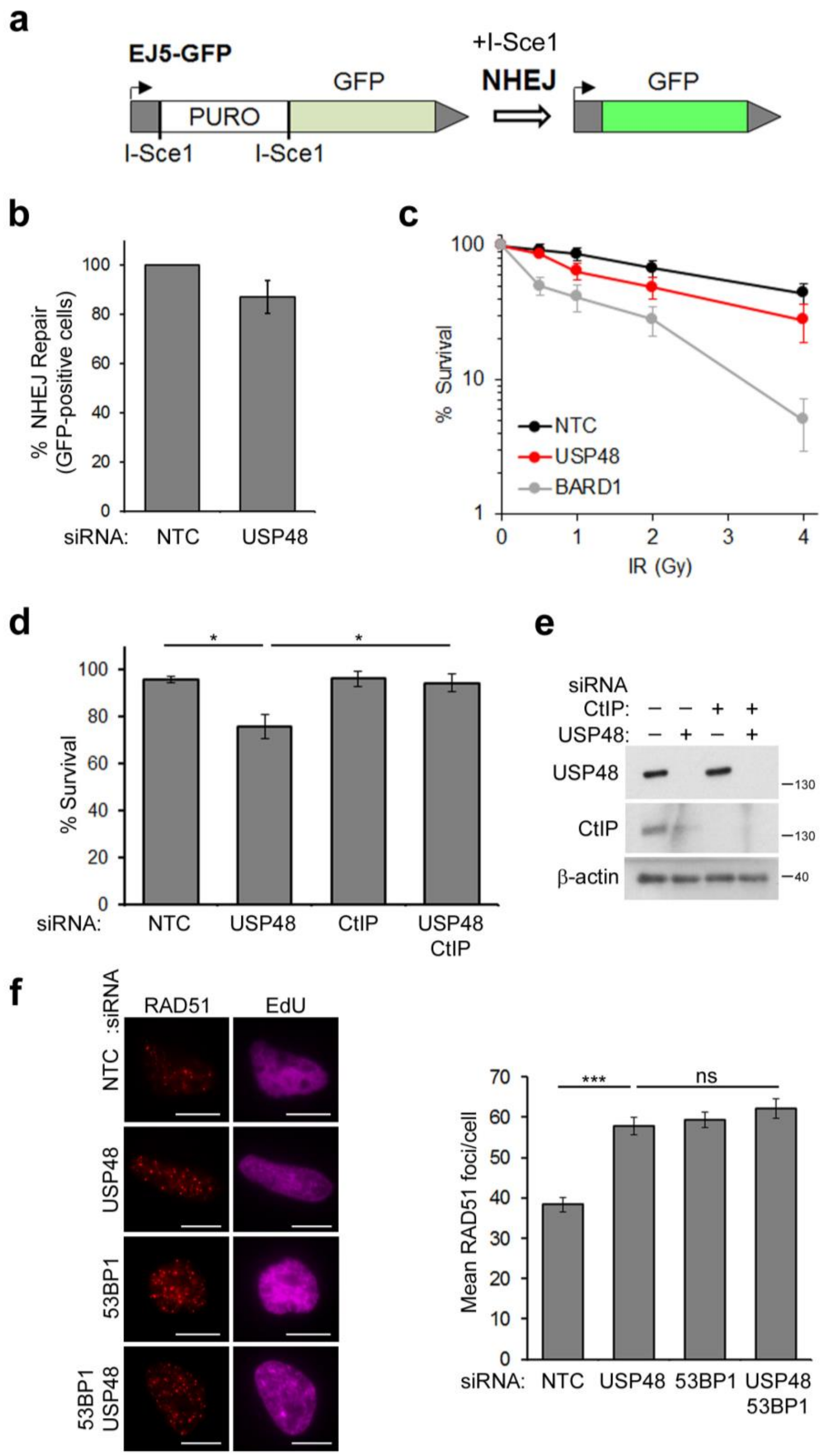

Supplementary Figure 8

USP48-depletion causes a mild defect in NHEJ

a. Illustration of the integrated NHEJ assay.

b. Cells treated with non-targeting control (NTC) and USP48 siRNA were transfected with RFP and *SCE-1* expressing plasmids. GFP-positive cells were normalised to RFP-transfection efficiency and %-repair is given compared to NTC. Graph shows mean, n=5, error bars are s.e.m.

c. Colony survival of HeLa cells depleted for USP48, BARD1 or control (NTC) siRNA and treated with ionising irradiation (IR). Graph shows mean % survival normalised to untreated controls, n=4, error bars are s.e.m.

d. Colony survival of HeLa cells depleted for USP48, CtIP and both USP48 and CtIP. Cells were treated with 1 Gy IR before plating out at limiting dilutions and grown for 10-14 days to form colonies. Graph shows mean % survival normalised to untreated controls, n=3, error bars are s.e.m. * p<0.05 Student's T-test.

e**.** Western blot of siRNA treated lysates probed for USP48 and CtIP.

f. USP48 knockdown increases Rad51 foci formation to the same extent as 53BP1 loss and is epistatic with 53BP1 loss. RAD51 foci formation was measured in S-phase (EdU positive) HeLa cells depleted for USP48, 53BP1 or both. Cells were fixed at 2 hr post-5 Gy irradiation. The images (left) show RAD51 and EdU staining, Scale bars 10 µm. The graph (right) shows quantification of mean RAD51 foci per S-phase cell (n=115 cells, error bars are s.e.m, *** p<0.005 Student's T-test).

Supplementary Figure 9

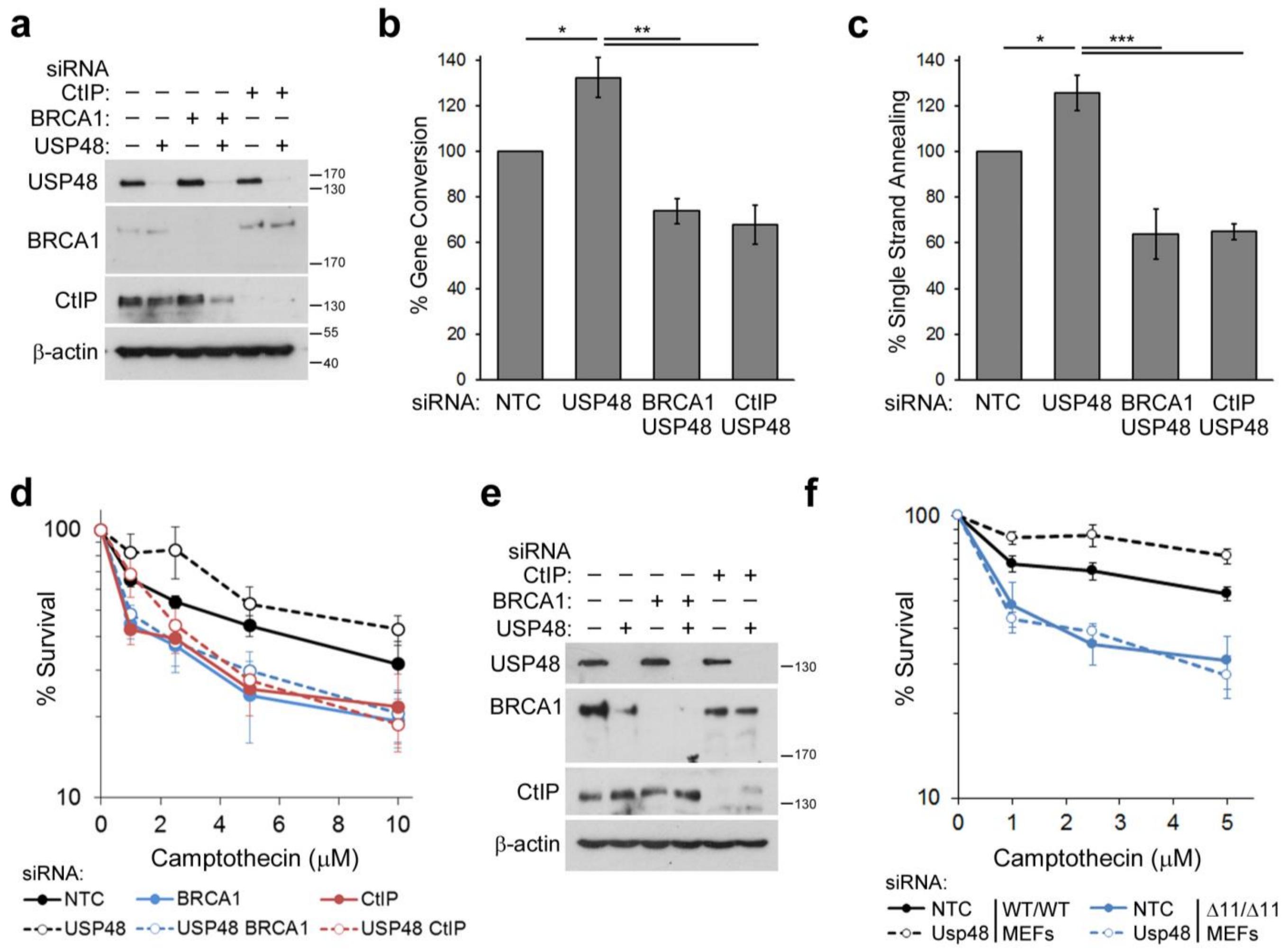

Supplementary Figure 9
USP48 loss does not confer a survival benefit in BRCA1-defective cells

a. Western blot of siRNA treated lysates from U20S-DR3-reporter cells probed for USP48, BRCA1 and CtIP.

b & c. Increased Gene Conversion (GC) and Single Strand Annealing (SSA) measures seen on USP48 depletion require BRCA1 and CtIP. GC and SSA assays were undertaken as described in Fig 5a and b) in cells treated with siRNA against non-targeting control (NTC), USP48 or USP48 together with BRCA1 or CtIP targeting sequences. GFP-positive cells were normalised to RFP-transfection efficiency. %-repair is given compared to NTC. Graph shows mean, n=5, error bars are s.e.m.

d. Camptothecin colony survival curves of HeLa cells depleted for USP48, BRCA1 or CtIP individual and USP48 with BRCA1 or CtIP. Graph shows mean % survival normalised to untreated controls, n=4, error bars are s.e.m.

e. Western blot shows protein expression levels in HeLa cells following siRNA depletion as indicated.

f. Camptothecin colony survival curves of *WT* and *Brca1*$^{\Delta11/\Delta11}$ MEFs treated with non-targeting control siRNA (NTC) or Usp48 siRNA. Graph shows mean % survival normalised to untreated controls, n=4, error bars are s.e.m.
